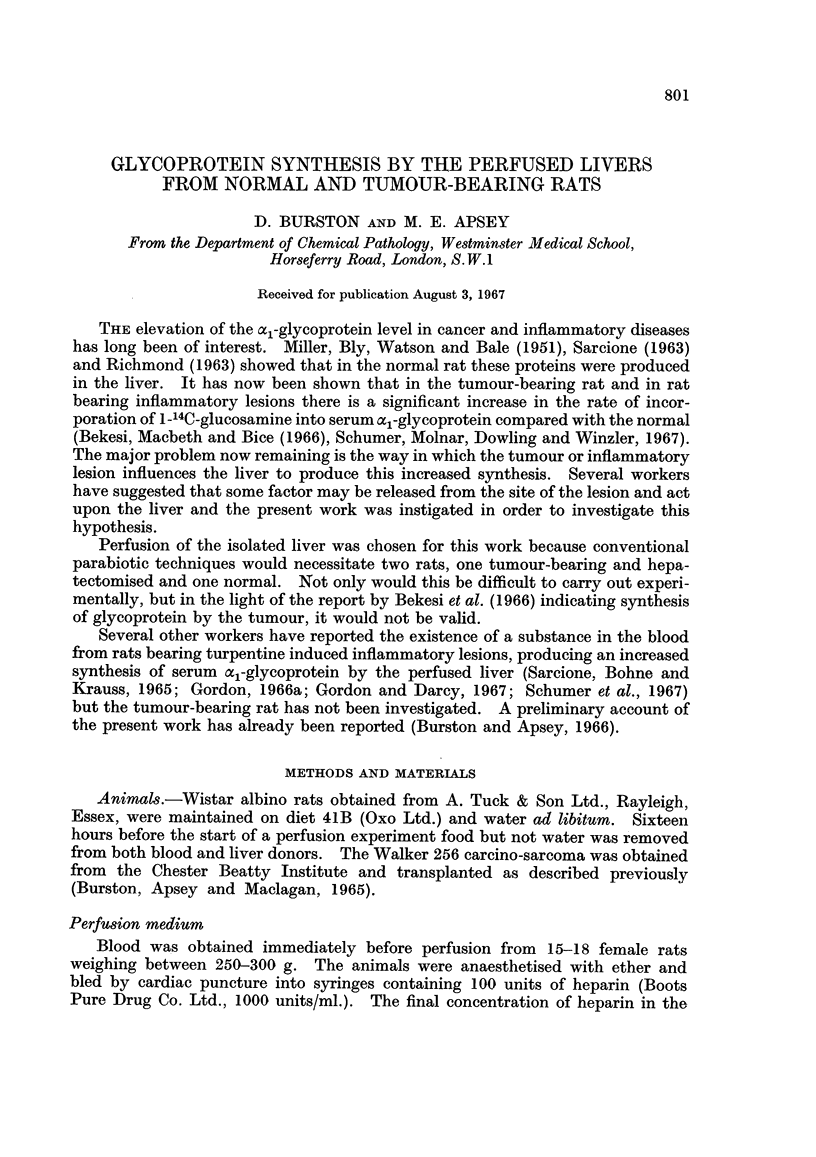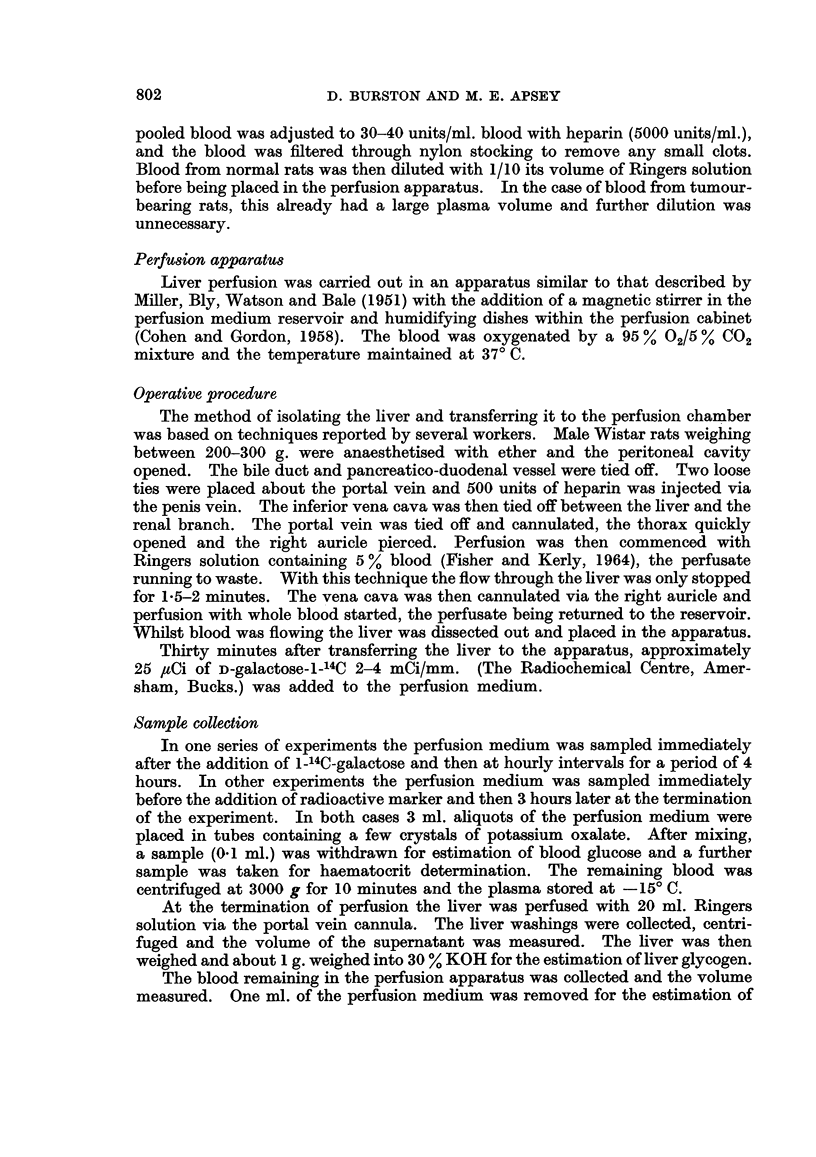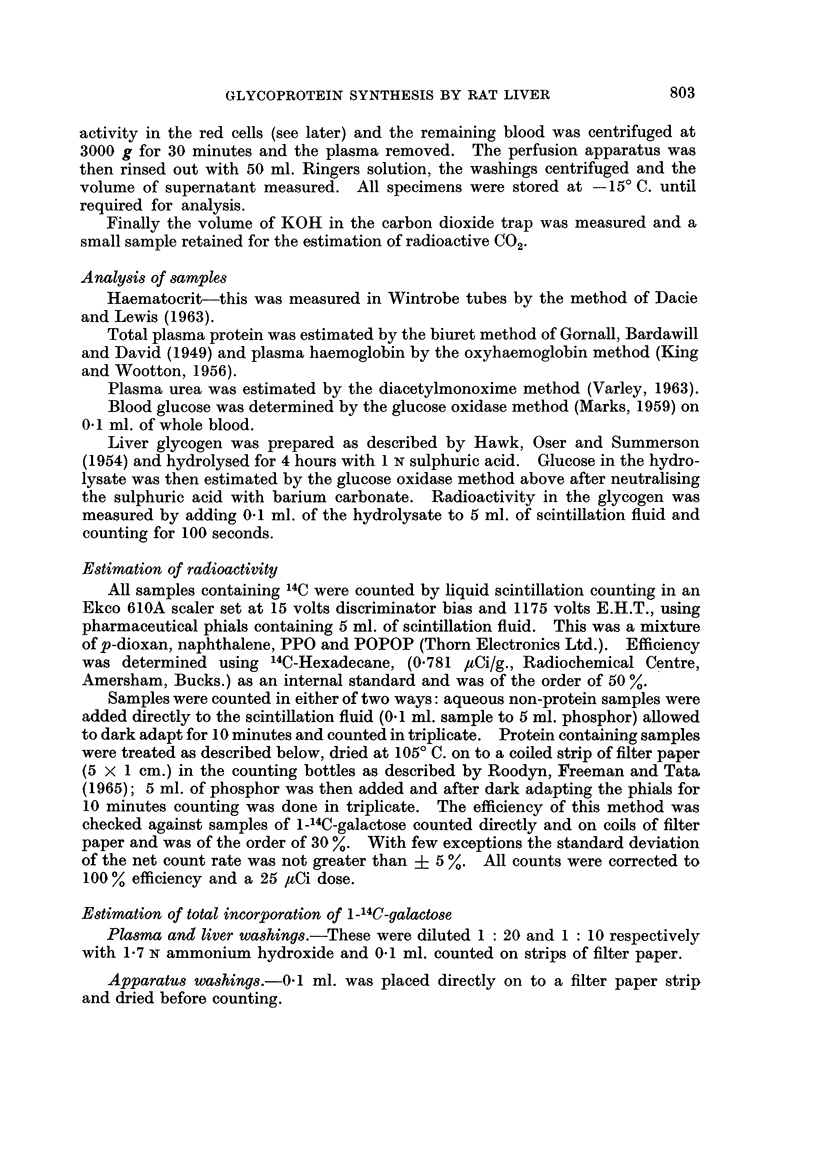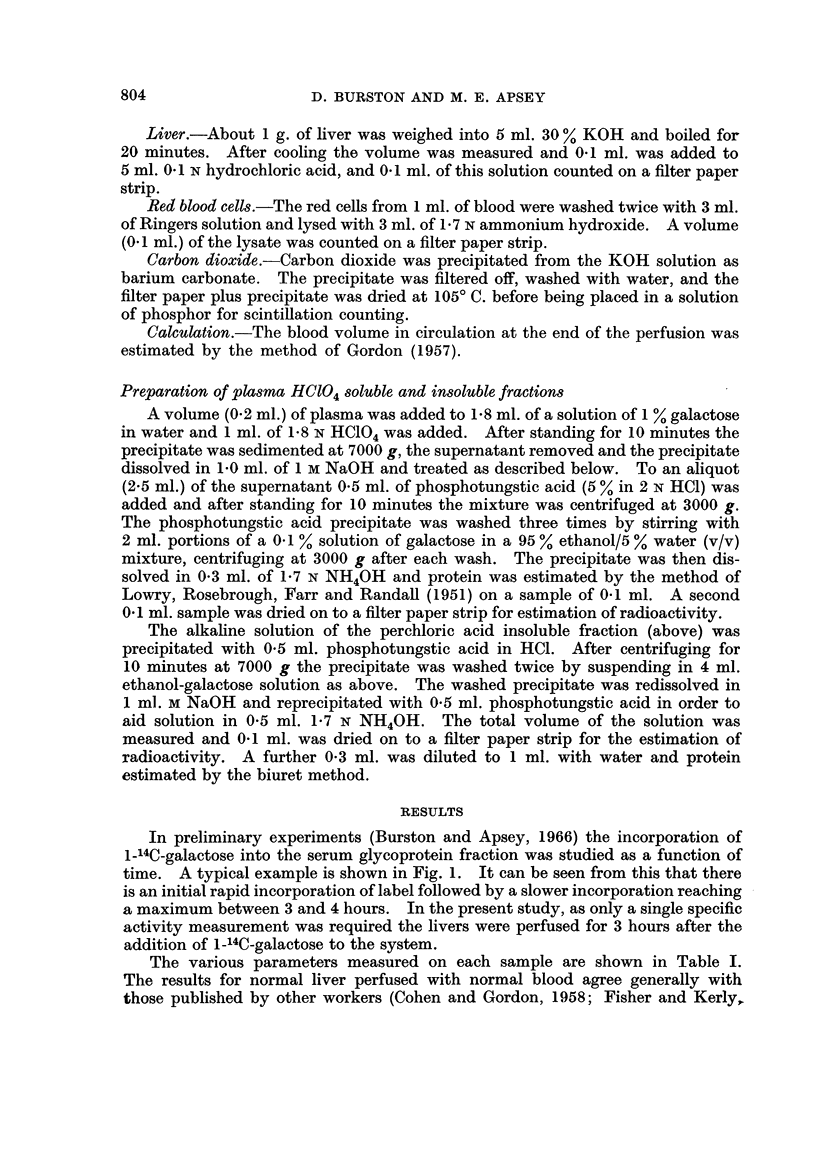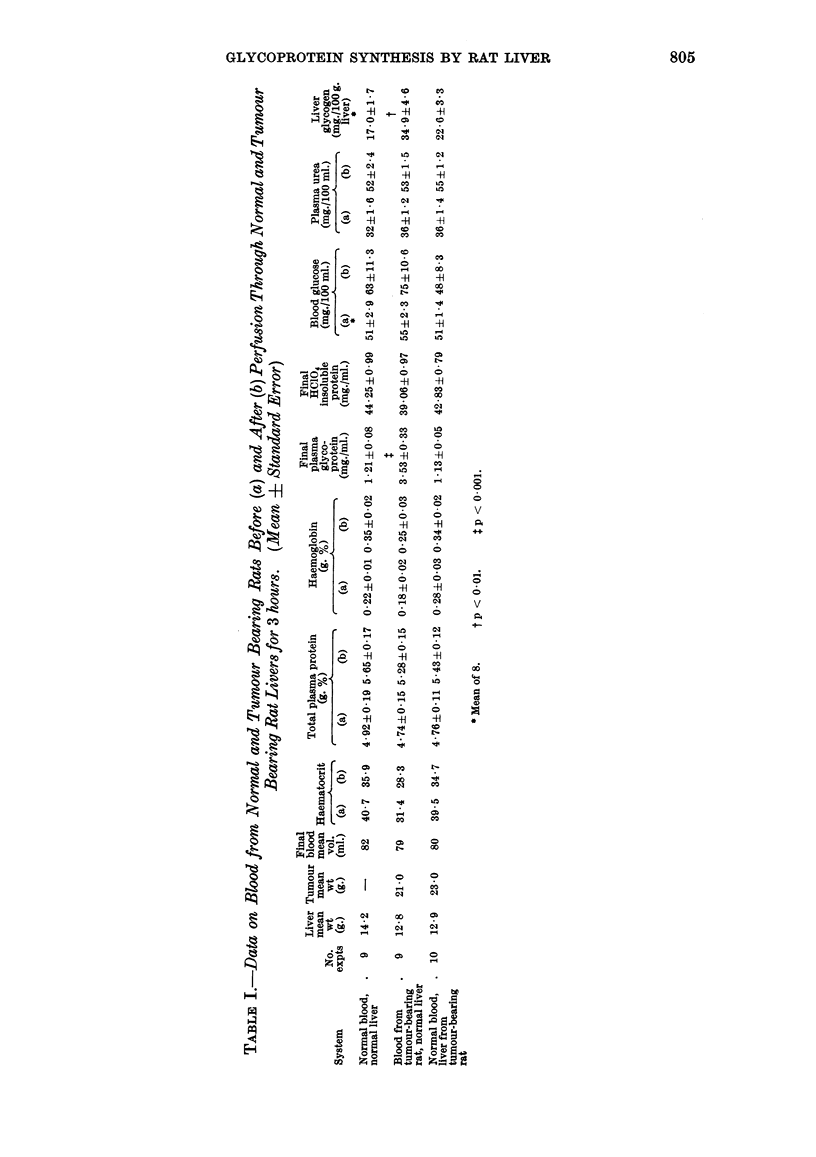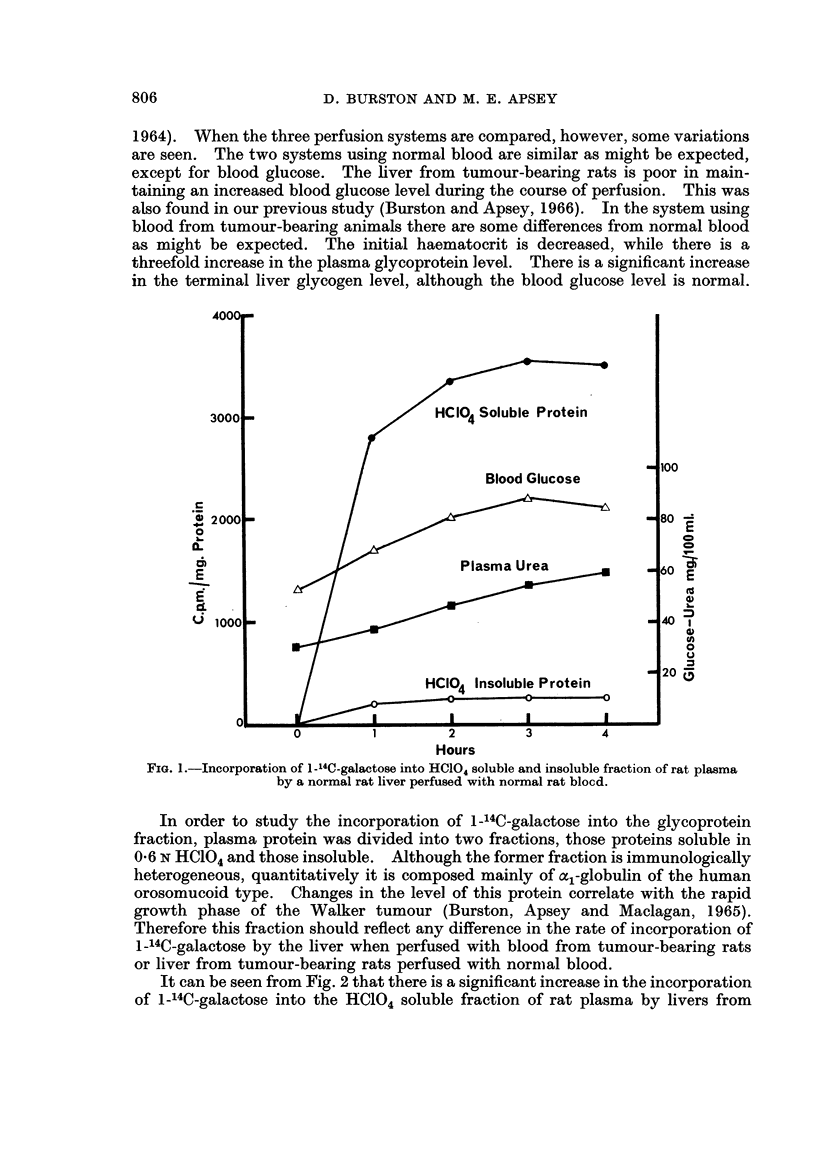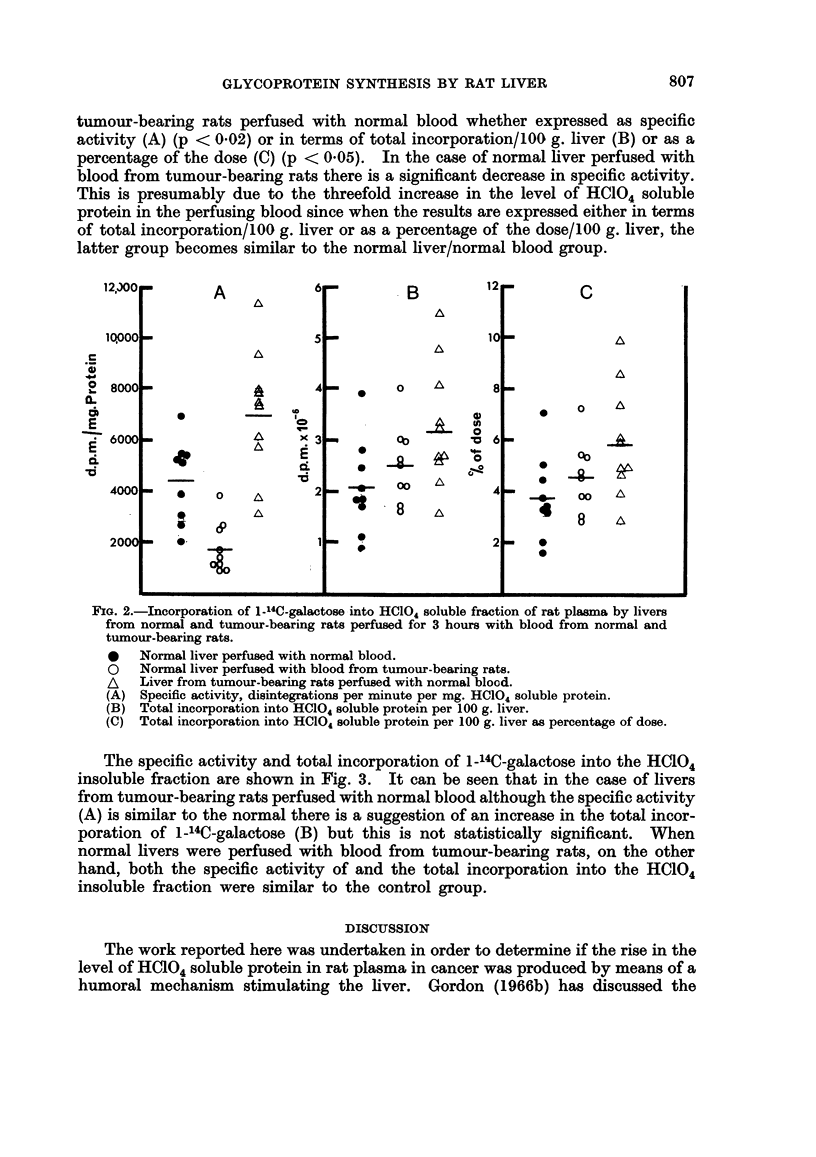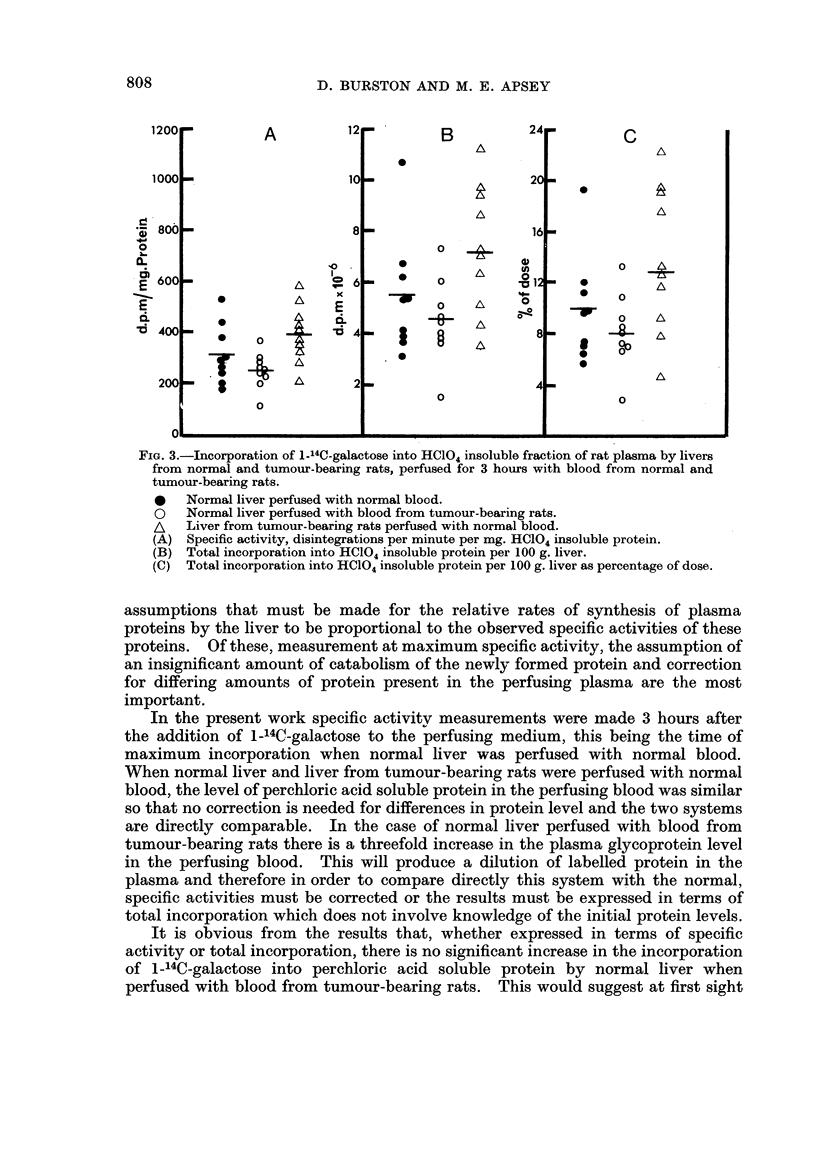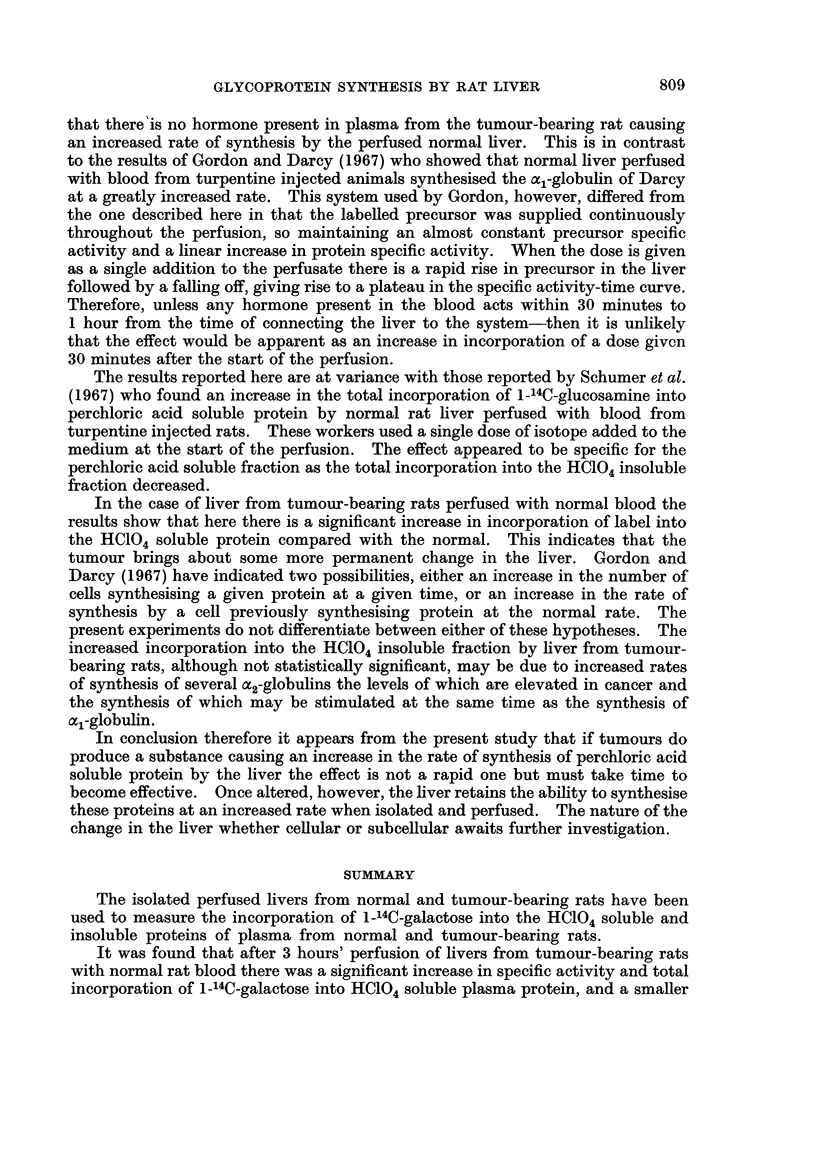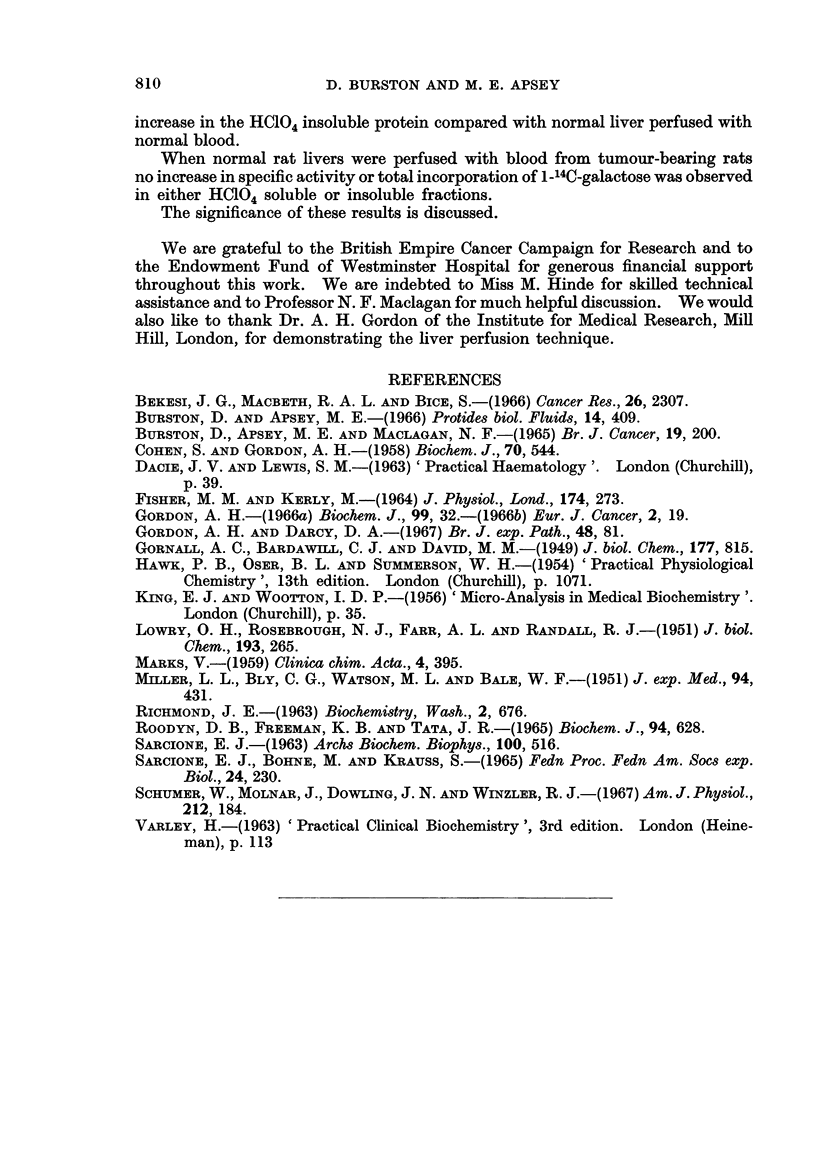# Glycoprotein synthesis by the perfused livers from normal and tumour-bearing rats.

**DOI:** 10.1038/bjc.1967.94

**Published:** 1967-12

**Authors:** D. Burston, M. E. Apsey


### GLYCOPROTEIN SYNTHESIS BY THE PERFUSED LIVERS FROM NORMAL AND TUMOUR-BEARING RATS

D. BURSTON AND M. E. APSEY

*From the Department of Chemical Pathology, Westminster Medical School, Horseferry Road, London, S.W.1*

Received for publication August 3, 1967

THE elevation of the $\alpha_1$-glycoprotein level in cancer and inflammatory diseases has long been of interest. Miller, Bly, Watson and Bale (1951), Sarcione (1963) and Richmond (1963) showed that in the normal rat these proteins were produced in the liver. It has now been shown that in the tumour-bearing rat and in rat bearing inflammatory lesions there is a significant increase in the rate of incorporation of 1-$^{14}$C-glucosamine into serum $\alpha_1$-glycoprotein compared with the normal (Bekesi, Macbeth and Bice (1966), Schumer, Molnar, Dowling and Winzler, 1967). The major problem now remaining is the way in which the tumour or inflammatory lesion influences the liver to produce this increased synthesis. Several workers have suggested that some factor may be released from the site of the lesion and act upon the liver and the present work was instigated in order to investigate this hypothesis.

Perfusion of the isolated liver was chosen for this work because conventional parabiotic techniques would necessitate two rats, one tumour-bearing and hepatectomised and one normal. Not only would this be difficult to carry out experimentally, but in the light of the report by Bekesi *et al.* (1966) indicating synthesis of glycoprotein by the tumour, it would not be valid.

Several other workers have reported the existence of a substance in the blood from rats bearing turpentine induced inflammatory lesions, producing an increased synthesis of serum $\alpha_1$-glycoprotein by the perfused liver (Sarcione, Bohne and Krauss, 1965; Gordon, 1966a; Gordon and Darcy, 1967; Schumer *et al.*, 1967) but the tumour-bearing rat has not been investigated. A preliminary account of the present work has already been reported (Burston and Apsey, 1966).

### METHODS AND MATERIALS

*Animals.*—Wistar albino rats obtained from A. Tuck & Son Ltd., Rayleigh, Essex, were maintained on diet 41B (Oxo Ltd.) and water *ad libitum.* Sixteen hours before the start of a perfusion experiment food but not water was removed from both blood and liver donors. The Walker 256 carcino-sarcoma was obtained from the Chester Beatty Institute and transplanted as described previously (Burston, Apsey and Maclagan, 1965).

### *Perfusion medium*

Blood was obtained immediately before perfusion from 15–18 female rats weighing between 250–300 g. The animals were anaesthetised with ether and bled by cardiac puncture into syringes containing 100 units of heparin (Boots Pure Drug Co. Ltd., 1000 units/ml.). The final concentration of heparin in the pooled blood was adjusted to 30–40 units/ml. blood with heparin (5000 units/ml.), and the blood was filtered through nylon stocking to remove any small clots. Blood from normal rats was then diluted with 1/10 its volume of Ringers solution before being placed in the perfusion apparatus. In the case of blood from tumour-bearing rats, this already had a large plasma volume and further dilution was unnecessary.

*Perfusion apparatus*

Liver perfusion was carried out in an apparatus similar to that described by Miller, Bly, Watson and Bale (1951) with the addition of a magnetic stirrer in the perfusion medium reservoir and humidifying dishes within the perfusion cabinet (Cohen and Gordon, 1958). The blood was oxygenated by a 95% $O_2$/5% $CO_2$ mixture and the temperature maintained at 37° C.

*Operative procedure*

The method of isolating the liver and transferring it to the perfusion chamber was based on techniques reported by several workers. Male Wistar rats weighing between 200–300 g. were anaesthetised with ether and the peritoneal cavity opened. The bile duct and pancreatico-duodenal vessel were tied off. Two loose ties were placed about the portal vein and 500 units of heparin was injected via the penis vein. The inferior vena cava was then tied off between the liver and the renal branch. The portal vein was tied off and cannulated, the thorax quickly opened and the right auricle pierced. Perfusion was then commenced with Ringers solution containing 5% blood (Fisher and Kerly, 1964), the perfusate running to waste. With this technique the flow through the liver was only stopped for 1·5–2 minutes. The vena cava was then cannulated via the right auricle and perfusion with whole blood started, the perfusate being returned to the reservoir. Whilst blood was flowing the liver was dissected out and placed in the apparatus.

Thirty minutes after transferring the liver to the apparatus, approximately 25 $\mu$Ci of D-galactose-1-$^{14}$C 2–4 mCi/mm. (The Radiochemical Centre, Amersham, Bucks.) was added to the perfusion medium.

*Sample collection*

In one series of experiments the perfusion medium was sampled immediately after the addition of 1-$^{14}$C-galactose and then at hourly intervals for a period of 4 hours. In other experiments the perfusion medium was sampled immediately before the addition of radioactive marker and then 3 hours later at the termination of the experiment. In both cases 3 ml. aliquots of the perfusion medium were placed in tubes containing a few crystals of potassium oxalate. After mixing, a sample (0·1 ml.) was withdrawn for estimation of blood glucose and a further sample was taken for haematocrit determination. The remaining blood was centrifuged at 3000 *g* for 10 minutes and the plasma stored at −15° C.

At the termination of perfusion the liver was perfused with 20 ml. Ringers solution via the portal vein cannula. The liver washings were collected, centrifuged and the volume of the supernatant was measured. The liver was then weighed and about 1 g. weighed into 30% KOH for the estimation of liver glycogen.

The blood remaining in the perfusion apparatus was collected and the volume measured. One ml. of the perfusion medium was removed for the estimation of activity in the red cells (see later) and the remaining blood was centrifuged at 3000 *g* for 30 minutes and the plasma removed. The perfusion apparatus was then rinsed out with 50 ml. Ringers solution, the washings centrifuged and the volume of supernatant measured. All specimens were stored at $-15°$ C. until required for analysis.

Finally the volume of KOH in the carbon dioxide trap was measured and a small sample retained for the estimation of radioactive $CO_2$.

*Analysis of samples*

Haematocrit—this was measured in Wintrobe tubes by the method of Dacie and Lewis (1963).

Total plasma protein was estimated by the biuret method of Gornall, Bardawill and David (1949) and plasma haemoglobin by the oxyhaemoglobin method (King and Wootton, 1956).

Plasma urea was estimated by the diacetylmonoxime method (Varley, 1963).

Blood glucose was determined by the glucose oxidase method (Marks, 1959) on 0·1 ml. of whole blood.

Liver glycogen was prepared as described by Hawk, Oser and Summerson (1954) and hydrolysed for 4 hours with 1 N sulphuric acid. Glucose in the hydrolysate was then estimated by the glucose oxidase method above after neutralising the sulphuric acid with barium carbonate. Radioactivity in the glycogen was measured by adding 0·1 ml. of the hydrolysate to 5 ml. of scintillation fluid and counting for 100 seconds.

*Estimation of radioactivity*

All samples containing $^{14}C$ were counted by liquid scintillation counting in an Ekco 610A scaler set at 15 volts discriminator bias and 1175 volts E.H.T., using pharmaceutical phials containing 5 ml. of scintillation fluid. This was a mixture of *p*-dioxan, naphthalene, PPO and POPOP (Thorn Electronics Ltd.). Efficiency was determined using $^{14}C$-Hexadecane, (0·781 $\mu$Ci/g., Radiochemical Centre, Amersham, Bucks.) as an internal standard and was of the order of 50 %.

Samples were counted in either of two ways: aqueous non-protein samples were added directly to the scintillation fluid (0·1 ml. sample to 5 ml. phosphor) allowed to dark adapt for 10 minutes and counted in triplicate. Protein containing samples were treated as described below, dried at 105° C. on to a coiled strip of filter paper ($5 \times 1$ cm.) in the counting bottles as described by Roodyn, Freeman and Tata (1965); 5 ml. of phosphor was then added and after dark adapting the phials for 10 minutes counting was done in triplicate. The efficiency of this method was checked against samples of 1-$^{14}C$-galactose counted directly and on coils of filter paper and was of the order of 30 %. With few exceptions the standard deviation of the net count rate was not greater than $\pm$ 5 %. All counts were corrected to 100 % efficiency and a 25 $\mu$Ci dose.

*Estimation of total incorporation of* 1-$^{14}$*C-galactose*

*Plasma and liver washings.*—These were diluted 1 : 20 and 1 : 10 respectively with 1·7 N ammonium hydroxide and 0·1 ml. counted on strips of filter paper.

*Apparatus washings.*—0·1 ml. was placed directly on to a filter paper strip and dried before counting.

*Liver.*—About 1 g. of liver was weighed into 5 ml. 30% KOH and boiled for 20 minutes. After cooling the volume was measured and 0·1 ml. was added to 5 ml. 0·1 N hydrochloric acid, and 0·1 ml. of this solution counted on a filter paper strip.

*Red blood cells.*—The red cells from 1 ml. of blood were washed twice with 3 ml. of Ringers solution and lysed with 3 ml. of 1·7 N ammonium hydroxide. A volume (0·1 ml.) of the lysate was counted on a filter paper strip.

*Carbon dioxide.*—Carbon dioxide was precipitated from the KOH solution as barium carbonate. The precipitate was filtered off, washed with water, and the filter paper plus precipitate was dried at 105° C. before being placed in a solution of phosphor for scintillation counting.

*Calculation.*—The blood volume in circulation at the end of the perfusion was estimated by the method of Gordon (1957).

### *Preparation of plasma $HClO_4$ soluble and insoluble fractions*

A volume (0·2 ml.) of plasma was added to 1·8 ml. of a solution of 1% galactose in water and 1 ml. of 1·8 N $HClO_4$ was added. After standing for 10 minutes the precipitate was sedimented at 7000 *g*, the supernatant removed and the precipitate dissolved in 1·0 ml. of 1 M NaOH and treated as described below. To an aliquot (2·5 ml.) of the supernatant 0·5 ml. of phosphotungstic acid (5% in 2 N HCl) was added and after standing for 10 minutes the mixture was centrifuged at 3000 *g*. The phosphotungstic acid precipitate was washed three times by stirring with 2 ml. portions of a 0·1% solution of galactose in a 95% ethanol/5% water (v/v) mixture, centrifuging at 3000 *g* after each wash. The precipitate was then dissolved in 0·3 ml. of 1·7 N $NH_4OH$ and protein was estimated by the method of Lowry, Rosebrough, Farr and Randall (1951) on a sample of 0·1 ml. A second 0·1 ml. sample was dried on to a filter paper strip for estimation of radioactivity.

The alkaline solution of the perchloric acid insoluble fraction (above) was precipitated with 0·5 ml. phosphotungstic acid in HCl. After centrifuging for 10 minutes at 7000 *g* the precipitate was washed twice by suspending in 4 ml. ethanol-galactose solution as above. The washed precipitate was redissolved in 1 ml. M NaOH and reprecipitated with 0·5 ml. phosphotungstic acid in order to aid solution in 0·5 ml. 1·7 N $NH_4OH$. The total volume of the solution was measured and 0·1 ml. was dried on to a filter paper strip for the estimation of radioactivity. A further 0·3 ml. was diluted to 1 ml. with water and protein estimated by the biuret method.

### RESULTS

In preliminary experiments (Burston and Apsey, 1966) the incorporation of 1-$^{14}$C-galactose into the serum glycoprotein fraction was studied as a function of time. A typical example is shown in Fig. 1. It can be seen from this that there is an initial rapid incorporation of label followed by a slower incorporation reaching a maximum between 3 and 4 hours. In the present study, as only a single specific activity measurement was required the livers were perfused for 3 hours after the addition of 1-$^{14}$C-galactose to the system.

The various parameters measured on each sample are shown in Table I. The results for normal liver perfused with normal blood agree generally with those published by other workers (Cohen and Gordon, 1958; Fisher and Kerly,

TABLE I.—*Data on Blood from Normal and Tumour Bearing Rats Before (a) and After (b) Perfusion Through Normal and Tumour Bearing Rat Livers for 3 hours. (Mean ± Standard Error)*

| System | No. expts | Liver mean wt (g.) | Tumour mean wt (g.) | Final blood mean vol. (ml.) | Haematocrit (a) | Haematocrit (b) | Total plasma protein (g. %) (a) | Total plasma protein (g. %) (b) | Haemoglobin (g. %) (a) | Haemoglobin (g. %) (b) | Final plasma glyco-protein (mg./ml.) | Final $HClO_4$ insoluble protein (mg./ml.) | Blood glucose (mg./100 ml.) (a) * | Blood glucose (mg./100 ml.) (b) | Plasma urea (mg./100 ml.) (a) | Plasma urea (mg./100 ml.) (b) | Liver glycogen (mg./100 g. liver) * |
|---|---|---|---|---|---|---|---|---|---|---|---|---|---|---|---|---|---|
| Normal blood, normal liver | 9 | 14·2 | — | 82 | 40·7 | 35·9 | 4·92±0·19 | 5·65±0·17 | 0·22±0·01 | 0·35±0·02 | 1·21±0·08 | 44·25±0·99 | 51±2·9 | 63±11·3 | 32±1·6 | 52±2·4 | 17·0±1·7 |
| Blood from tumour-bearing rat, normal liver | 9 | 12·8 | 21·0 | 79 | 31·4 | 28·3 | 4·74±0·15 | 5·28±0·15 | 0·18±0·02 | 0·25±0·03 | ‡ 3·53±0·33 | 39·06±0·97 | 55±2·3 | 75±10·6 | 36±1·2 | 53±1·5 | † 34·9±4·6 |
| Normal blood, liver from tumour-bearing rat | 10 | 12·9 | 23·0 | 80 | 39·5 | 34·7 | 4·76±0·11 | 5·43±0·12 | 0·28±0·03 | 0·34±0·02 | 1·13±0·05 | 42·83±0·79 | 51±1·4 | 48±8·3 | 36±1·4 | 55±1·2 | 22·6±3·3 |

* Mean of 8. † $p < 0·01$. ‡ $p < 0·001$.

1964). When the three perfusion systems are compared, however, some variations are seen. The two systems using normal blood are similar as might be expected, except for blood glucose. The liver from tumour-bearing rats is poor in maintaining an increased blood glucose level during the course of perfusion. This was also found in our previous study (Burston and Apsey, 1966). In the system using blood from tumour-bearing animals there are some differences from normal blood as might be expected. The initial haematocrit is decreased, while there is a threefold increase in the plasma glycoprotein level. There is a significant increase in the terminal liver glycogen level, although the blood glucose level is normal.

FIG. 1.—Incorporation of 1-$^{14}$C-galactose into $HClO_4$ soluble and insoluble fraction of rat plasma by a normal rat liver perfused with normal rat blood.

In order to study the incorporation of 1-$^{14}$C-galactose into the glycoprotein fraction, plasma protein was divided into two fractions, those proteins soluble in 0·6 N $HClO_4$ and those insoluble. Although the former fraction is immunologically heterogeneous, quantitatively it is composed mainly of $\alpha_1$-globulin of the human orosomucoid type. Changes in the level of this protein correlate with the rapid growth phase of the Walker tumour (Burston, Apsey and Maclagan, 1965). Therefore this fraction should reflect any difference in the rate of incorporation of 1-$^{14}$C-galactose by the liver when perfused with blood from tumour-bearing rats or liver from tumour-bearing rats perfused with normal blood.

It can be seen from Fig. 2 that there is a significant increase in the incorporation of 1-$^{14}$C-galactose into the $HClO_4$ soluble fraction of rat plasma by livers from tumour-bearing rats perfused with normal blood whether expressed as specific activity (A) ($p < 0{\cdot}02$) or in terms of total incorporation/100 g. liver (B) or as a percentage of the dose (C) ($p < 0{\cdot}05$). In the case of normal liver perfused with blood from tumour-bearing rats there is a significant decrease in specific activity. This is presumably due to the threefold increase in the level of $HClO_4$ soluble protein in the perfusing blood since when the results are expressed either in terms of total incorporation/100 g. liver or as a percentage of the dose/100 g. liver, the latter group becomes similar to the normal liver/normal blood group.

FIG. 2.—Incorporation of 1-$^{14}$C-galactose into $HClO_4$ soluble fraction of rat plasma by livers from normal and tumour-bearing rats perfused for 3 hours with blood from normal and tumour-bearing rats.

● Normal liver perfused with normal blood.
○ Normal liver perfused with blood from tumour-bearing rats.
△ Liver from tumour-bearing rats perfused with normal blood.
(A) Specific activity, disintegrations per minute per mg. $HClO_4$ soluble protein.
(B) Total incorporation into $HClO_4$ soluble protein per 100 g. liver.
(C) Total incorporation into $HClO_4$ soluble protein per 100 g. liver as percentage of dose.

The specific activity and total incorporation of 1-$^{14}$C-galactose into the $HClO_4$ insoluble fraction are shown in Fig. 3. It can be seen that in the case of livers from tumour-bearing rats perfused with normal blood although the specific activity (A) is similar to the normal there is a suggestion of an increase in the total incorporation of 1-$^{14}$C-galactose (B) but this is not statistically significant. When normal livers were perfused with blood from tumour-bearing rats, on the other hand, both the specific activity of and the total incorporation into the $HClO_4$ insoluble fraction were similar to the control group.

### DISCUSSION

The work reported here was undertaken in order to determine if the rise in the level of $HClO_4$ soluble protein in rat plasma in cancer was produced by means of a humoral mechanism stimulating the liver. Gordon (1966b) has discussed the

FIG. 3.—Incorporation of 1-$^{14}$C-galactose into $HClO_4$ insoluble fraction of rat plasma by livers from normal and tumour-bearing rats, perfused for 3 hours with blood from normal and tumour-bearing rats.

● Normal liver perfused with normal blood.
○ Normal liver perfused with blood from tumour-bearing rats.
△ Liver from tumour-bearing rats perfused with normal blood.
(A) Specific activity, disintegrations per minute per mg. $HClO_4$ insoluble protein.
(B) Total incorporation into $HClO_4$ insoluble protein per 100 g. liver.
(C) Total incorporation into $HClO_4$ insoluble protein per 100 g. liver as percentage of dose.

assumptions that must be made for the relative rates of synthesis of plasma proteins by the liver to be proportional to the observed specific activities of these proteins. Of these, measurement at maximum specific activity, the assumption of an insignificant amount of catabolism of the newly formed protein and correction for differing amounts of protein present in the perfusing plasma are the most important.

In the present work specific activity measurements were made 3 hours after the addition of 1-$^{14}$C-galactose to the perfusing medium, this being the time of maximum incorporation when normal liver was perfused with normal blood. When normal liver and liver from tumour-bearing rats were perfused with normal blood, the level of perchloric acid soluble protein in the perfusing blood was similar so that no correction is needed for differences in protein level and the two systems are directly comparable. In the case of normal liver perfused with blood from tumour-bearing rats there is a threefold increase in the plasma glycoprotein level in the perfusing blood. This will produce a dilution of labelled protein in the plasma and therefore in order to compare directly this system with the normal, specific activities must be corrected or the results must be expressed in terms of total incorporation which does not involve knowledge of the initial protein levels.

It is obvious from the results that, whether expressed in terms of specific activity or total incorporation, there is no significant increase in the incorporation of 1-$^{14}$C-galactose into perchloric acid soluble protein by normal liver when perfused with blood from tumour-bearing rats. This would suggest at first sight that there is no hormone present in plasma from the tumour-bearing rat causing an increased rate of synthesis by the perfused normal liver. This is in contrast to the results of Gordon and Darcy (1967) who showed that normal liver perfused with blood from turpentine injected animals synthesised the $\alpha_1$-globulin of Darcy at a greatly increased rate. This system used by Gordon, however, differed from the one described here in that the labelled precursor was supplied continuously throughout the perfusion, so maintaining an almost constant precursor specific activity and a linear increase in protein specific activity. When the dose is given as a single addition to the perfusate there is a rapid rise in precursor in the liver followed by a falling off, giving rise to a plateau in the specific activity-time curve. Therefore, unless any hormone present in the blood acts within 30 minutes to 1 hour from the time of connecting the liver to the system—then it is unlikely that the effect would be apparent as an increase in incorporation of a dose given 30 minutes after the start of the perfusion.

The results reported here are at variance with those reported by Schumer *et al.* (1967) who found an increase in the total incorporation of 1-$^{14}$C-glucosamine into perchloric acid soluble protein by normal rat liver perfused with blood from turpentine injected rats. These workers used a single dose of isotope added to the medium at the start of the perfusion. The effect appeared to be specific for the perchloric acid soluble fraction as the total incorporation into the $HClO_4$ insoluble fraction decreased.

In the case of liver from tumour-bearing rats perfused with normal blood the results show that here there is a significant increase in incorporation of label into the $HClO_4$ soluble protein compared with the normal. This indicates that the tumour brings about some more permanent change in the liver. Gordon and Darcy (1967) have indicated two possibilities, either an increase in the number of cells synthesising a given protein at a given time, or an increase in the rate of synthesis by a cell previously synthesising protein at the normal rate. The present experiments do not differentiate between either of these hypotheses. The increased incorporation into the $HClO_4$ insoluble fraction by liver from tumour-bearing rats, although not statistically significant, may be due to increased rates of synthesis of several $\alpha_2$-globulins the levels of which are elevated in cancer and the synthesis of which may be stimulated at the same time as the synthesis of $\alpha_1$-globulin.

In conclusion therefore it appears from the present study that if tumours do produce a substance causing an increase in the rate of synthesis of perchloric acid soluble protein by the liver the effect is not a rapid one but must take time to become effective. Once altered, however, the liver retains the ability to synthesise these proteins at an increased rate when isolated and perfused. The nature of the change in the liver whether cellular or subcellular awaits further investigation.

### SUMMARY

The isolated perfused livers from normal and tumour-bearing rats have been used to measure the incorporation of 1-$^{14}$C-galactose into the $HClO_4$ soluble and insoluble proteins of plasma from normal and tumour-bearing rats.

It was found that after 3 hours' perfusion of livers from tumour-bearing rats with normal rat blood there was a significant increase in specific activity and total incorporation of 1-$^{14}$C-galactose into $HClO_4$ soluble plasma protein, and a smaller increase in the $HClO_4$ insoluble protein compared with normal liver perfused with normal blood.

When normal rat livers were perfused with blood from tumour-bearing rats no increase in specific activity or total incorporation of 1-$^{14}$C-galactose was observed in either $HClO_4$ soluble or insoluble fractions.

The significance of these results is discussed.

We are grateful to the British Empire Cancer Campaign for Research and to the Endowment Fund of Westminster Hospital for generous financial support throughout this work. We are indebted to Miss M. Hinde for skilled technical assistance and to Professor N. F. Maclagan for much helpful discussion. We would also like to thank Dr. A. H. Gordon of the Institute for Medical Research, Mill Hill, London, for demonstrating the liver perfusion technique.

### REFERENCES

BEKESI, J. G., MACBETH, R. A. L. AND BICE, S.—(1966) *Cancer Res.*, **26**, 2307.
BURSTON, D. AND APSEY, M. E.—(1966) *Protides biol. Fluids*, **14**, 409.
BURSTON, D., APSEY, M. E. AND MACLAGAN, N. F.—(1965) *Br. J. Cancer*, **19**, 200.
COHEN, S. AND GORDON, A. H.—(1958) *Biochem. J.*, **70**, 544.
DACIE, J. V. AND LEWIS, S. M.—(1963) ‘ Practical Haematology ’. London (Churchill), p. 39.
FISHER, M. M. AND KERLY, M.—(1964) *J. Physiol., Lond.*, **174**, 273.
GORDON, A. H.—(1966*a*) *Biochem. J.*, **99**, 32.—(1966*b*) *Eur. J. Cancer*, **2**, 19.
GORDON, A. H. AND DARCY, D. A.—(1967) *Br. J. exp. Path.*, **48**, 81.
GORNALL, A. C., BARDAWILL, C. J. AND DAVID, M. M.—(1949) *J. biol. Chem.*, **177**, 815.
HAWK, P. B., OSER, B. L. AND SUMMERSON, W. H.—(1954) ‘ Practical Physiological Chemistry ’, 13th edition. London (Churchill), p. 1071.
KING, E. J. AND WOOTTON, I. D. P.—(1956) ‘ Micro-Analysis in Medical Biochemistry ’. London (Churchill), p. 35.
LOWRY, O. H., ROSEBROUGH, N. J., FARR, A. L. AND RANDALL, R. J.—(1951) *J. biol. Chem.*, **193**, 265.
MARKS, V.—(1959) *Clinica chim. Acta.*, **4**, 395.
MILLER, L. L., BLY, C. G., WATSON, M. L. AND BALE, W. F.—(1951) *J. exp. Med.*, **94**, 431.
RICHMOND, J. E.—(1963) *Biochemistry, Wash.*, **2**, 676.
ROODYN, D. B., FREEMAN, K. B. AND TATA, J. R.—(1965) *Biochem. J.*, **94**, 628.
SARCIONE, E. J.—(1963) *Archs Biochem. Biophys.*, **100**, 516.
SARCIONE, E. J., BOHNE, M. AND KRAUSS, S.—(1965) *Fedn Proc. Fedn Am. Socs exp. Biol.*, **24**, 230.
SCHUMER, W., MOLNAR, J., DOWLING, J. N. AND WINZLER, R. J.—(1967) *Am. J. Physiol.*, **212**, 184.
VARLEY, H.—(1963) ‘ Practical Clinical Biochemistry ’, 3rd edition. London (Heineman), p. 113